# Dose optimization of inhaled tigecycline in humans to overcome inherent adverse events and maximize bacterial clearance using a physiologically-based pharmacokinetic modeling approach

**DOI:** 10.3389/ftubr.2026.1762713

**Published:** 2026-03-26

**Authors:** Hyunseo Park, Amarinder Singh, Ashish Srivastava, Bhargavi Thalluri, Christelle J. Mathieu, Paridhi Gupta, Camron M. Pearce, Malik Zohaib Ali, Ilham M. Alshiraihi, Sara E. Maloney Norcross, Anthony J. Hickey, Mercedes Gonzalez-Juarrero, Bernd Meibohm

**Affiliations:** 1 Department of Pharmaceutical Sciences, College of Pharmacy, University of Tennessee Health Science Center, Memphis, TN, United States; 2 Mycobacteria Research Laboratories, Department of Microbiology, Immunology, and Pathology, Colorado State University, Fort Collins, CO, United States; 3 Department of Engineering and Advanced Technology, RTI International, Durham, NC, United States

**Keywords:** inhalation therapy, *Mycobacterium abscessus*, non-tuberculous mycobacteria, physiologically-based pharmacokinetic modeling, tigecycline

## Abstract

**Introduction:**

Intrapulmonary delivery of tigecycline has been highlighted as an optimal strategy for enhancing local drug concentrations at the site of infection in the treatment of *Mycobacterium abscessus* pulmonary infections. Therefore, determining an appropriate inhaled dose is imperative to optimize therapeutic use of tigecycline. In this study, we aimed at establishing a human dose rationale for inhaled tigecycline by leveraging various preclinical experimental datasets through physiologically-based pharmacokinetic (PBPK) modeling.

**Methods:**

The PBPK model developed to predict plasma and target-site exposure of inhaled tigecycline and to relate these exposures to adverse event thresholds was derived from plasma and tissue concentration-time courses of *in vivo* mouse studies. Following inter-species scaling, the predictive performance of the model was qualified by comparing model-based simulations with experimental data in rats and literature reports in humans, thereby demonstrating its applicability across species. The final human PBPK model was utilized to predict tigecycline exposure in plasma and major organs of interest, thereby establishing the clinical utility of tigecycline inhaled dosing.

**Results:**

Using model-based simulations, we predicted the longitudinal exposure profiles of tigecycline in the systemic circulation, the epithelial lining fluid (ELF) in the lungs as site of antibacterial activity, and other major organs following inhalation under clinically relevant conditions. Intrapulmonary aerosol dosing using the currently approved intravenous dose of tigecycline was predicted to result in significantly lower plasma exposure compared to the gastrointestinal adverse event threshold reported in the literature (AUC_0−24_ 6.87 mg·h/L). Additionally, simulated steady-state bone concentrations remained below a threshold which has been previously defined as a steady-state trough concentration leading to bone toxicity in rats. For efficacy, intrapulmonary aerosol administration produced markedly higher peak concentrations in ELF than intravenous dosing, and the simulated exposure in ELF exceeded effective exposure levels identified in murine infection models of *Mycobacterium abscessus*.

**Discussion:**

Our findings indicate that compared to conventional intravenous infusion, inhaled tigecycline offers an improved safety margin and enhances bacterial killing. Based on simulations for multiple dosing scenarios in humans, a dose of 135 mg given every third day by intrapulmonary delivery would result in ELF, bone, and plasma exposures that effectively balance efficacy and safety.

## 1 Introduction

Opportunistic pulmonary infections caused by *Mycobacterium abscessus* primarily occur in specific patient populations, such as individuals with cystic fibrosis, chronic obstructive pulmonary disease, or bronchiectasis (1, 2), and are a leading cause of mortality in these groups (3–5). The low treatment success rate in patients infected with *M. abscessus* is largely attributed to the pathogen's intrinsic drug resistance (6) and biofilm formation that restricts penetration of antibiotics to the site of infection (7). These mechanisms significantly hinder the activity of most antibiotics, regardless of whether they are administered alone or in combination. Consequently, overcoming these defense mechanisms requires achieving sufficiently high drug concentrations at the local target site to ensure anti-bacterial activity.

Tigecycline (TGC), a third generation glycylcycline antibiotic administered via intravenous (IV) infusion, has demonstrated favorable clinical efficacy against *M. abscessus* infections (8). However, due to its narrow therapeutic window, TGC at the approved intravenous (IV) standard dose of 50 mg given twice daily frequently induces gastrointestinal adverse events (9). Additionally, severe side effects such as bone discoloration and growth inhibition have been reported, requiring caution in its use (10). The incidence of these adverse events is closely linked to systemic exposure (11), making targeted delivery by inhaled administration an attractive alternative for treating pulmonary infections. By delivering the drug directly to the lungs, the inhaled TGC results in effective local drug concentrations while minimizing systemic toxicity. Pre-clinical studies (12) and a recently published clinical case report (13) support the therapeutic potential of inhaled TGC. However, current clinical evidence remains very limited. Moreover, previous studies primarily focused on efficacy rather than pharmacokinetics (PK). As a result, the dose-exposure related parameters for inhaled TGC have not yet been explored. Therefore, to optimize clinical use of inhaled TGC, it is essential to quantitatively assess both target site and systemic exposure as rational basis for developing appropriate dosing regimens.

For this purpose, a quantitative modeling framework predicting drug exposure under various conditions is required. In this manuscript, we describe development and application of a physiologically based pharmacokinetic (PBPK) modeling approach to simulate systemic, target-site, and toxicity-related organ exposures following inhaled administration of TGC in humans based on preclinical data in rodents. Our model also accounts for the non-linear plasma protein binding of TGC, a key factor previously described as influencing drug distribution and efficacy (14–16). By integrating available human PK and tissue distribution data after IV administration from the literature, we refined the model to enhance its predictive performance and clinical applicability. Finally, model-based simulations under various scenarios, combined with clinically established systemic exposure-based adverse event thresholds, were utilized to propose an optimal dosing regimen for inhaled TGC in humans. It is anticipated that our model-based approach will serve as a foundation for optimizing dosing strategies of inhaled TGC therapy.

## 2 Materials and methods

### 2.1 *In vivo* pharmacokinetic studies in rodents

*In vivo* PK and tissue distribution studies were conducted in C57BL/6 mice (Charles River, Wilmington, MA) using various doses (10, 50, 70, and 100 mg/kg) and administration routes, including IV, subcutaneous (SC), and intrapulmonary aerosol (IPA). For IPA administration, a FMJ-250 high-pressure syringe device (Penn Century, Philadelphia, PA, United States) with an attached MicroSprayer (MicroSprayer, model IA-C; Penn Century) was used as previously described (12, 17). Under anesthesia, the MicroSprayer tip was inserted into the upper trachea, and 50 μL of drug solution dissolved in normal saline was sprayed into the lungs. Depending on the study, groups of three or four mice were randomly assigned to each time point, without distinction between male and female subjects. At designated time points, 0, 0.083, 0.25, 0.5, 1, 2, 3, 6, 8, and 24 h post-administration, animals were euthanized by isoflurane inhalation followed by thoracotomy, and blood and tissues were collected. Bronchoalveolar lavage fluid (BALF) was collected by flushing the lungs three times with 200 μL of saline solution using a 22G catheter (Becton Dickinson, Sparks, MD), inserted through the trachea. Additionally, lungs, kidney, liver, and spleen samples were collected and stored at −80°C until analysis.

For the *in vivo* rat PK and tissue distribution study, six rats (3 males/3 females) with jugular vein catheter were assigned to the IV or SC study group. Each group received a single dose of 30 mg/kg TGC, and blood samples were collected at multiple time points (0.083, 0.25, 0.5, 1, 3, 6, and 10 h). Three rats were sacrificed for tissue sampling at 2 h post dose and the other three at the 10-h sampling time point. Lungs were flushed with 400 μL of saline solution three times and BALF was collected using 24G catheters inserted through the trachea. As in the mouse study, lungs, kidney, liver, and spleen samples were collected after euthanasia by isoflurane inhalation followed by thoracotomy and stored at −80°C until analysis. All animal datasets obtained here and used for PBPK model development are summarized in Table 1.

**Table 1:** Summary of clinical and non-clinical pharmacokinetic and tissue distribution data of TGC from the literature, and performed experiments used for the development of the multi-species PBPK model of TGC. The use of each dataset is indicated using capital letters: N, Q, D, and P, which represent “not used,” “model qualification,” “model development,” and “re-parameterization,” respectively.

**Author**	**Species/strains**	**Disease conditions**	**Available dataset**	**Dosing regimen**	**Route**	**Use of dataset**	**Reference**
Muralidharan et al.	Human	Healthy adults	Plasma	12.5–300 mg	IV infusion	N	(29)
Bulik et al.	Human	Diabetic patients with chronic wound infections	Plasma, skin†	100 mg LD + 50 mg bid x 3 times	IV infusion	N	(20)
Gotfried et al.	Human	Healthy adults	Plasma, ELF, AV cells	100 mg LD + 50 mg bid x 7 times	IV infusion	Q	(58)
Bhattacharya et al.	Human	Patients undergoing elective orthopedic surgery	Serum, bone	100 mg LD + 50 mg bid x 2 times	IV infusion	Q	(59)
Pascale et al.	Human	Critically ill patients with severe infections	Plasma, ELF	200 mg LD + 100 mg bid x 7 times	IV infusion	Q	(51)
Conte et al.	Human	Healthy adults	Plasma, ELF, AV cells	100 mg LD + 50 mg bid x 6 times	IV infusion	Q	(60)
Korth-Bradley et al.	Human ‡	Patients with cirrhosis	Plasma	100 mg single dose	IV infusion	N	(61)
Rodvold et al.	Human	Subjects undergoing surgical or medical procedures	Plasma, lung, bone, colon, SF, CSF, GB, Bile	100 mg single dose	IV infusion	Q, P	(19)
Hoffmann et al.	Human §	Healthy adults	Plasma, urine, feces	100 mg LD + 50 mg bid x 5 times	IV infusion	N	(62)
Guo et al.	Human	Critically ill patients, diagnosed with hospital-acquired pneumonia	Plasma, ELF^#^	200 mg LD + 100 mg bid ≥ 6 times	IV infusion	N	(63)
Murphy et al.	Wistar rats	Endocarditis model	Serum	7, 20, 40 mg/kg single dose	SC	Q, P	(28)
Goessens et al.	specified-pathogen-free RP/AEur/RijHsd albino rats	*Klebsiella Pneumonia* infected	Plasma	6.25, 12.5 mg/kg single dose, and qd x 5 times	IP	N	(64)
Experimental	SD-Rats	Healthy	Plasma, ELF, lung, liver, kidney	30 mg/kg, single dose	SC	Q	N/A
Experimental	SD-Rats	Healthy	Plasma, ELF, lung, liver, kidney	30 mg/kg, single dose	IV bolus	Q	N/A
Experimental	C57BL/6 mice	Healthy	Plasma, ELF, lung, liver, kidney	10 mg/kg, single dose	IV bolus	D	N/A
Experimental	C57BL/6 mice	Healthy	Plasma, ELF, lung, liver, kidney	10 mg/kg, qd x 5 times	IV bolus	D	N/A
Experimental	C57BL/6 mice	Healthy	Plasma, ELF, lung, liver, kidney	10 mg/kg, single dose	SC	D	N/A
Experimental	C57BL/6 mice	Healthy	Plasma, ELF, lung, liver, kidney	10 mg/kg, qd x 5 times	SC	D	N/A
Experimental	C57BL/6 mice	Healthy	Plasma, ELF, lung, liver, kidney	10, 50, 100 mg/kg, single dose	IPA	D	N/A
Experimental	C57BL/6 mice	Healthy	Plasma, ELF, lung, liver, kidney	10, 50, 70 mg/kg, qd x 5 times	IPA	D	N/A

†, Microdialysis study; ‡,Hepatic impairment study; §, Mass balance study; ^#^, No sampling time information.

### 2.2 Quantification of TGC in biological matrices

Tissue samples were thawed and homogenized with four times their volume of phosphate buffered saline (PBS, 10 mM, pH 7.4). A 50 μL aliquot of tissue homogenate, BALF or plasma sample was mixed with acetonitrile containing 0.1% formic acid for protein precipitation. After centrifugation, a 5 μL aliquot of the supernatant was injected into an LC-MS/MS triple quadruple mass spectrometer (AB Sciex, Foster City, CA) equipped with electrospray ionization in multiple reaction monitoring mode using the mass transfers of m/z 586.3 → 513.2 for TGC and m/z 595.5 → 514.3 for deuterated d9-TGC used as an internal standard. The chromatography mobile phase consisted of water and acetonitrile/methanol 75:25 (v/v), each containing 0.2% formic acid. The analytes were eluted on a C18 column (4.6 x 50 mm, 3.5 μm, Waters, Milford, MA) with gradient methodology. Duplicate calibration curves covering the observed concentration range were used to quantify concentrations of each measurement, and internal quality control was provided in each analytical run with five different concentrations of TGC spiked into blank matrix samples, which were scattered between unknown samples in each analytical run. The lower limit of quantification was 2.93 ng/mL, with acceptable accuracy and precision deviating less than 20%. To determine the epithelial lining fluid (ELF) concentration, urea concentrations were quantified in BALF samples and corresponding plasma samples using a commercial kit (BioAssay Systems, Hayward, CA). The TGC concentration obtained from the LC-MS/MS analysis of BALF was then corrected based on the urea assay results (18).

### 2.3 Physiologically based pharmacokinetic model development in mice

A PBPK model was initially developed using longitudinal plasma and organ concentration-time profiles obtained from the available *in vivo* mouse studies to characterize the distribution of TGC in major organs. TGC concentration data were obtained by randomly assigning individual mice to different time points, with one observation per each time point. The generated values were subsequently pooled into one analysis and treated as the concentration-time profile of a single virtual animal. The overall model development process in mice is described below.

**Figure 1:**
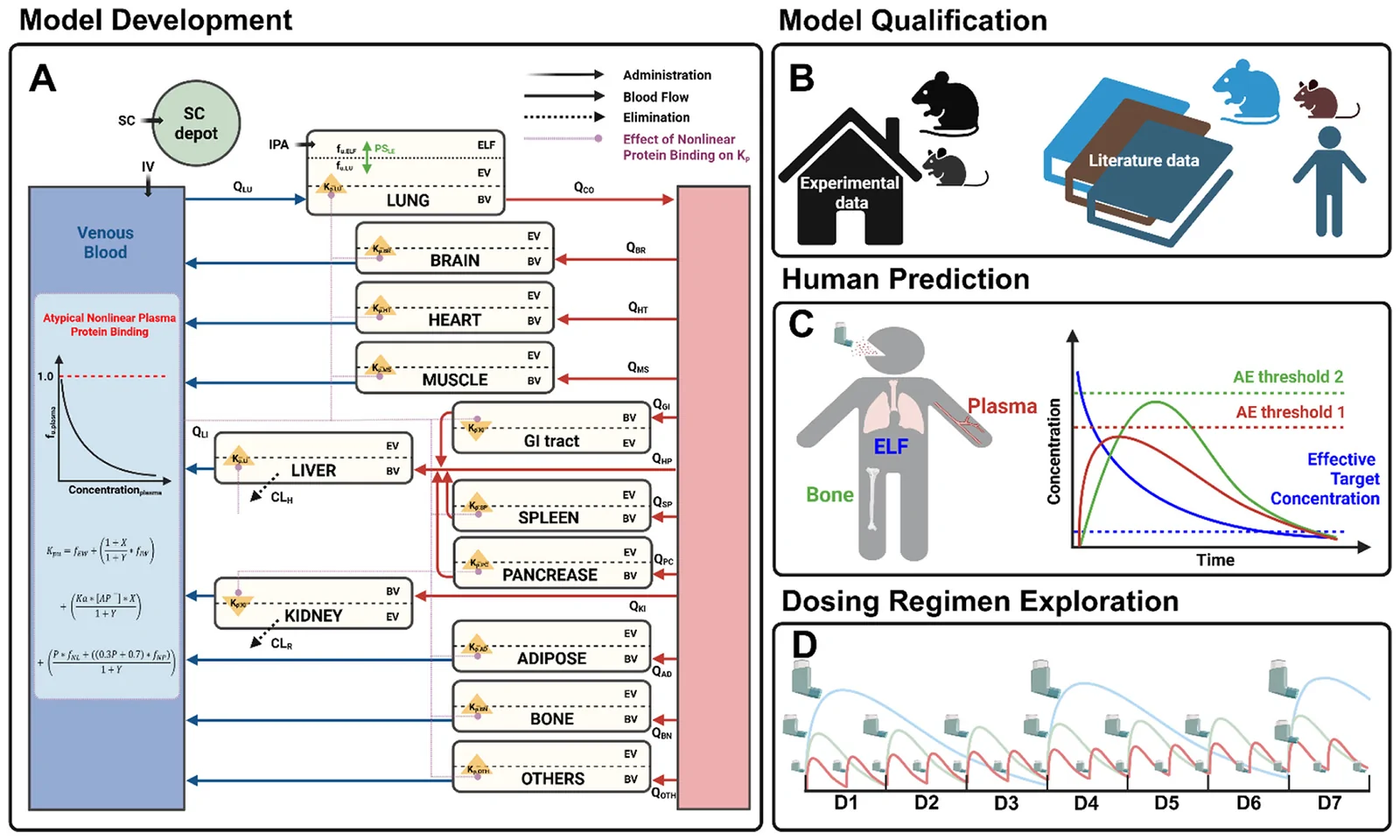
Human IPA dose prediction strategies: The final PBPK model **(A)** was developed in mice, subsequently scaled up to higher species (rat, human), and qualified through comparison with literature-reported and experimentally generated datasets **(B)**. The final human PBPK model was then used to predict the pharmacokinetics following IPA administration **(C)** in plasma and tissues of interest (ELF, bone), enabling comparison of exposure across various dosing regimens and the selection of exposure optimized IPA dosing strategies **(D)**.

The anatomical structure and inter-organ blood flow relationships incorporated into the model are illustrated in the schematic representation of the PBPK model (Figure 1). Given the rapid equilibrium and parallel terminal elimination between plasma and the organs observed in the *in vivo* study (19, 20), and that TGC shows rapid uptake through cell walls (21), a perfusion rate-limited PBPK model was adopted (22). Therefore, each organ compartment was divided into a blood vascular (BV) and an extravascular (EV) compartment, assuming instantaneous equilibrium between the two compartments. To represent whole-body distribution, the global PBPK model incorporated a total of 11 organs, including one that lumped multiple tissues with similar distribution characteristics together. These organs were based on the underlying physiology connected to the systemic venous and arterial blood pools. For all organs, except for the liver and lungs, blood inlet concentrations to the organ (C_blood.in_) was defined as the concentration in arterial blood (C_arterial.blood_), while blood outlet concentration (C_blood.out_) represented the concentration in the venous blood leaving the organ (C_organ_/K_p.organ_), where K_p.organ_ represents the organ-to-plasma partition coefficient. The final model structure also accounted for the impact of TGC's unique tissue distribution characteristics based on its non-linear plasma protein binding. The detailed differential equations for the PBPK model and the corresponding model code are provided in the Supplementary material. The physiological parameters of mice used for model development were obtained from the literature (23–26) and are summarized in Table S1 of the Supplementary material.

### 2.4 Model scale-up and qualification

The PBPK model developed based on mouse data was scaled up to predict rat and human PK and tissue distribution. The physiological parameters of rats and humans used for the scaling process were obtained from the literature (23–26) and are summarized in Table S1 in the Supplementary material. Among these parameters, the intercompartmental clearance between the lung and ELF, PS_LE_, was scaled allometrically using an exponent of 0.67, according to previous literature (27). For humans, accurate prediction of bone concentrations following IPA administration and their association with the probability of adverse events is of clinical importance. Therefore, physiological parameters such as the composition of the bone and the relative density of each bone region reported in the literature were also taken into consideration (23). Simulation profiles derived from the scaled-up models were compared with experimentally generated (rat) and literature-based digitized datasets (rat and human) to ensure their correctness and to overall assess the robustness and predictability of the PBPK modeling approach. The datasets used for model qualification and their sources are summarized in Table 1. To ensure relevance, only the datasets that simultaneously reported concentrations both in plasma and organs associated with efficacy and/or adverse events were utilized.

The available absorption-related data for SC administration in mice were limited to a single dose of 10 mg/kg, which was insufficient to capture a potential dose-dependency of the absorption process as previously reported in rats (28). Therefore, following the initial qualification step, the absorption rate constant in rats (k_a.sc.rat_) was re-parameterized using digitized data from the literature (28). Similarly, the model-based predictions in human lung tissue and ELF indicated slight over prediction of exposure. To address this, the partition coefficient adjustment value in human lung was re-parameterized using the digitized human datasets obtained from clinical studies that reported pulmonary TGC concentration profiles (19). During this reparameterization process, all other parameters were fixed to their scaled-up values.

The final model was used to predict drug exposure in the systemic circulation as the driver for gastrointestinal adverse events, exposure at target sites associated with both, antibacterial activity (ELF) to treat *M. abscessus* pulmonary infection, and potential bone-related adverse events following intrapulmonary administration in humans.

### 2.5 Model-based simulations to investigate optimal dosing in humans following IPA administration

For the simulations in humans, it was assumed that TGC follows linear kinetics after intrapulmonary administration, based on findings from a Phase 1 clinical study where TGC exhibited highly linear pharmacokinetic profiles across a wide range of single and multiple doses (29). For safety evaluations, the steady-state plasma AUC_0 − 24_ (AUC_0 − 24.*ss*.*pl*_) previously shown to significantly increase the probability of GI-related adverse events in clinical trials (11), and the corresponding average and maximum concentrations in plasma at the steady-state (C_avg.ss.pl_ and C_max.ss.pl_, respectively) were used as systemic exposure-based safety thresholds for GI-related adverse events. In addition, using the scaled-up rat PBPK model, the bone concentration in rats following repeated IV administration of 20 mg/kg TGC, which is known to trigger adverse events as reported in the FDA label (30), was simulated (Figure S1 in the Supplementary material), and a trough concentration in bone at steady-state (C_trough.ss.bn_ 1 μg/mL) was assumed as the adverse event threshold for bone-associated toxicity.

For antibacterial efficacy, the minimum effective concentration in ELF as the site of antibacterial activity was defined as the simulated trough concentration in ELF at the steady-state (C_trough.ss.elf_ 0.15 μg/mL) following a dosing regimen of 25 mg BID IPA, which had demonstrated clinical efficacy in a case study in a patient with *M. abscessus* infection (13). Any doses that failed to meet this efficacy criteria, despite meeting the safety threshold, were excluded from final dose selection.

Based on these assumptions, we explored 1.) Intrapulmonary doses that achieve systemic exposure below the safety threshold for maximum tolerated exposure using the same dosing interval as applied in the previous clinical case study, 2.) The maximum dosing interval maintaining target site exposure above the efficacy threshold based on the doses identified in 1.), and 3.) The maximum intrapulmonary dose that could be administered with a maximum once-weekly (QW) interval while remaining within the safety and efficacy limits. The optimal dosing regimen for intrapulmonary administration was ultimately selected by considering both safety and efficacy criteria, and practical aspects such as feasible drug amount to be delivered in each single administration.

### 2.6 Modeling software and graphic tools

The PBPK model was developed using Monolix 2024R1 (Lixoft, Antony, France), where differential equations were encoded and model parameters were estimated. Model-based simulations following the scale-up from the base model developed in mice were performed using Simulx (Lixoft, Antony, France) and R version 4.4.3 with the rxode2 package (31). Plotting and graphical analyses of the simulated datasets were conducted using R version 4.4.3 (32) (R Foundation for Statistical Computing, Vienna, Austria) and RStudio 2024.12.1(33) (Rstudio Inc., Boston, MA), utilizing the ggplot2 package (34).

## 3 Results

### 3.1 PBPK base-model development in mice

Model parameters for the PBPK base model in mice were estimated using the plasma and tissue concentration data for TGC obtained from *in vivo* PK studies in mice, and the results are summarized in Table 2. The absorption rate constant (k_a.sc_) was estimated to be 6.46 h^−1^, indicating a very rapid TGC absorption process following SC administration in mice. This value is also supportive of the observed high SC TGC bioavailability (~100%) observed in this species. The intercompartmental clearance between the lung and ELF compartments (PS_LE_) was estimated as 0.95 mL/h, which, given that the apparent ELF fluid volume in mice is less than 2 μL (35), suggests rapid molecular transfer between these two compartments.

**Table 2:** Parameter estimates for the TGC PBPK model in mice, and scaled-up and re-parameterized values for rats and humans.

**Types**	**Model parameters**	**Unit**	**Population values**	**RSE (%)**	**CV (%)**	**RSE (%)**	**Sources**	**Descriptions**
Parameter estimates	k_a.sc_	1/hr	6.46	22.3	–	22.3	Estimated	The first order absorption rate constant post-SC administration
	PS_LE_	L/hr	0.00095	4.9	–	–	Estimated	Intercompartmental clearance between lung and ELF. Product of permeability (m/hr) and surface area (m^2^)
	K_p.liver.adj_		0.66	7.52	23.7	30	Estimated	Adjustment value for ‘organ to plasma partition coefficient of liver'
	K_p.spleen.adj_		0.65	11.4	63.4	13.4	Estimated	Adjustment value for ‘organ to plasma partition coefficient of spleen'
	K_p.kidney.adj_		0.44	9.56	44.3	16.6	Estimated	Adjustment value for ‘organ to plasma partition coefficient of kidney'
	K_p.lung.adj_		0.38	34.2	461	12.8	Estimated	Adjustment value for ‘organ to plasma partition coefficient of lung'
	K_p.others_		3.34	12.5			Estimated	Adjustment value for ‘organ to plasma partition coefficient of rumped organs'
	f_u.lung_		0.33	24.7	173	15.3	Estimated	Unbound fraction of tigecycline in lung
	F_IPA_		0.43	19.8	92.6	16.5	Estimated	Bioavailability of TGA following IPA administration
Scaled-up values	PS_LE.rat_	L/hr	0.0043	–	–	–	Calculated	Scaled-up value of *PS*_*LE*_ for rat
	PS_LE.hu_	L/hr	0.19	–	–	–	Calculated	Scaled-up value of *PS*_*LE*_ for human
Re-parameterized	k_a.sc.rat_		0.43	18	–	–	Estimated	Re-parameterized first-order absorption rate constant for SC administration
	ED_50.rat_	mg/kg	31.9	23.9	–	–	Estimated	Re-parameterized value for dose that gives half-maximal absorption rate constant
	K_p, lung.adj.hu_		0.085	17.3	–	–	Estimated	Re-parameterized adjustment value for ‘organ to plasma partition coefficient of lung'
	**Organs**	**Values**	**RSE (%)**	**Organs**	**Values**	**RSE (%)**	**Sources**	**Description**
Parameter estimates for proportional error terms	Plasma	0.65	5.31	Lung	0.9	22.3	Estimated	Fixed parameters for each variable that defines the magnitude of the proportional error
	Kidney	0.86	7.27	Spleen	0.68	6.46	Estimated	
	Liver	0.89	7.74	ELF	1	10.6	Estimated	

The individual organ partition coefficients K_p.organ.adj_ were estimated for each tissue and ranged from 0.38 to 0.66 across organs. These values required to describe the observed data were lower than those theoretically calculated using the Rodger and Rowland method (36) frequently applied in PBPK modeling under consideration of the non-linear plasma protein binding of TGC. The estimated unbound fraction in the lung (f_u.lung_) was 0.33, which is close to the concentration-dependent unbound fraction in plasma, ranging from 0.064 to 0.34 across concentrations from 0.75 to 25 μg/mL as measured in mouse plasma in our experiments. This plasma protein binding profile is comparable to that observed in rats and humans (29, 37, 38). The estimated bioavailability for the inhalation route (F_IPA_) was 43%, which is considered high compared to currently used inhalation technologies in humans, where for example glucocorticoids administered with dry powder inhalers typically achieve bioavailability in the range of 30–50% (39). The relative standard error values (RSE %), representing parameter uncertainty, were below 35% for all parameters, suggesting that the model-related parameters were identifiable and could generally be robustly estimated.

The model-predicted concentration-time profiles in plasma and tissues and their underlying experimental data are compared in Figure S2 in the Supplementary material. Model diagnostic plots [Figure S3, panels (A) and (B) in the Supplementary material] for observed vs. predicted concentrations showed a slight overprediction tendency, primarily in the IPA group. This tendency is likely attributed to increased variability in TGC concentration measurements across plasma and tissues in animals treated with the IPA doses, presumably due to increased variability in the IPA dose delivered to each animal. This increased inter-individual variability is also evident in the weighted residual plots [Figure S3, panels (C) and (D) in the Supplementary material], where data points from the IPA group showed notably positive residuals.

### 3.2 Model scale-up and qualification

**Figure 2:**
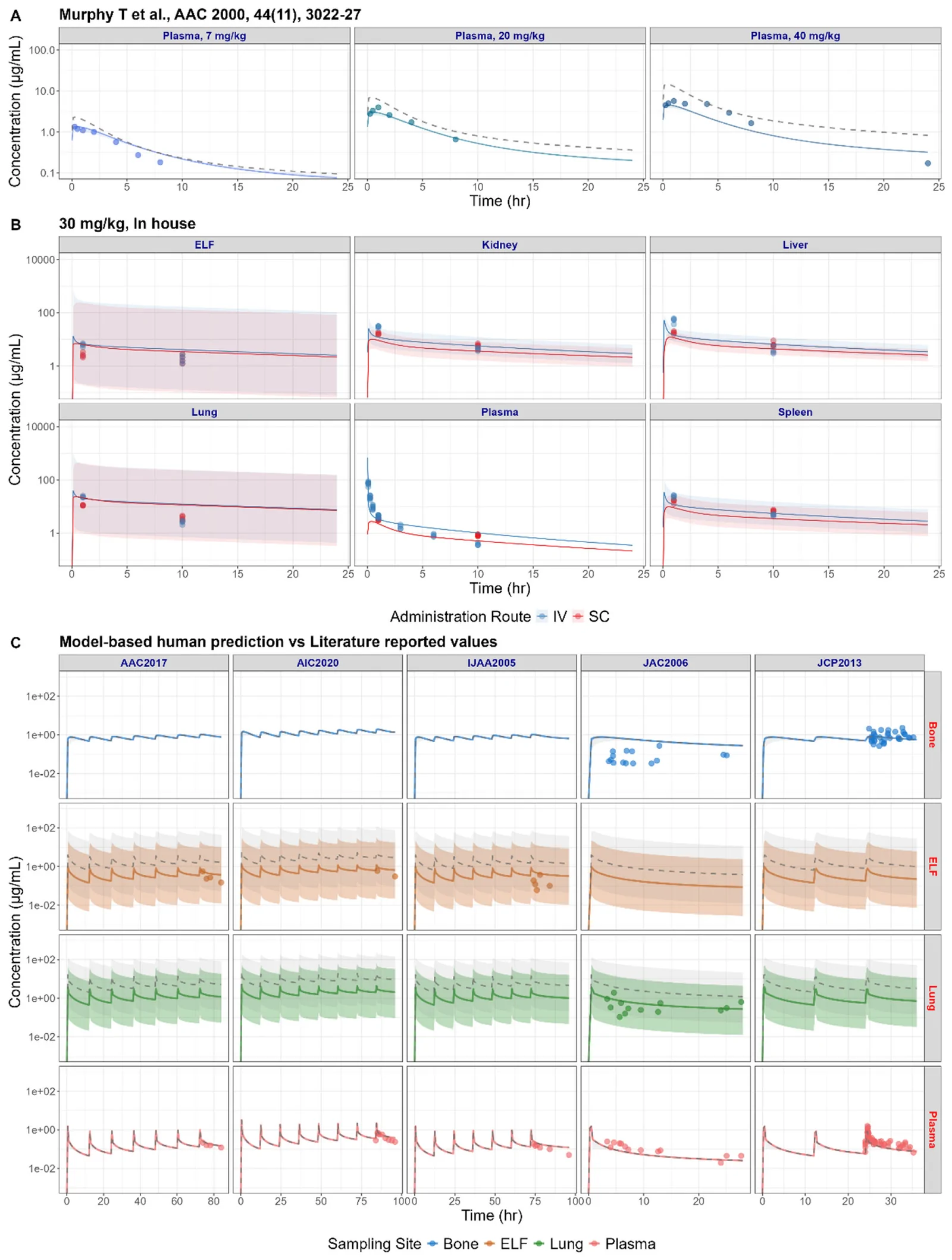
Model qualification results. Model-based simulation results were compared with literature-reported plasma concentration-time profiles across various dose levels **(A)** and experimentally obtained pharmacokinetic and multi-organ distribution datasets **(B)** in rat. Simulation profiles and 95% CI for human **(C)** were compared with various literature reported datasets as listed in Table 1. Solid and gray dashed lines represent the re-parameterized and original model prediction profiles, respectively, while the dots indicate clinically or experimentally observed values.

Following the parameter estimation step for the mouse PBPK model, it was scaled up to larger species including rats and humans. The model-based simulation profiles overlaid with the underlying experimental TGC concentration-time data in these species are presented in Figures 2A, B. The simulated profiles for rats and their 95% prediction intervals, generated utilizing the re-parameterized k_a.sc.rat_ and the dose for half-maximal absorption rate constant ED_50.rat_ from the model qualification step (Table 2), adequately captured the dose-dependent TGC absorption reported in the literature (28). They also described well the experimentally generated TGC concentrations in plasma and major organs following the administration 30 mg/kg IV and 30 mg/kg SC doses (Figure 2B).

The PBPK model scaled-up to humans only required re-parameterization of the partition coefficient adjustment value for lung (i.e., K_p.lung.adj.hu_) to reasonably describe the longitudinal plasma and organ concentration-time profiles of TGC reported in the literature across various IV dosing regimens and patient populations as shown in Figure 2C. The model parameters derived from this step were subsequently used to predict human plasma and tissue concentration-time profiles following IPA administration of TGC to facilitate optimal dose selection through model-based simulations.

### 3.3 Simulation of pharmacokinetic profiles in humans following intrapulmonary administration

Using the re-parameterized human PBPK model parameters obtained through the qualification step, time courses of TGC concentrations in plasma, bone, and ELF following IV administration of the clinical standard dosing regimen (100 mg loading dose followed by 50 mg BID administration) were simulated and compared to the concentration-time profiles in these organs after administration of the same dose (50 mg BID) or the efficacious dose from the clinical case report (25 mg BID) (13) using pulmonary delivery, thereby assuming that the TGC bioavailability for IPA administration is identical between mice and humans.

**Figure 3:**
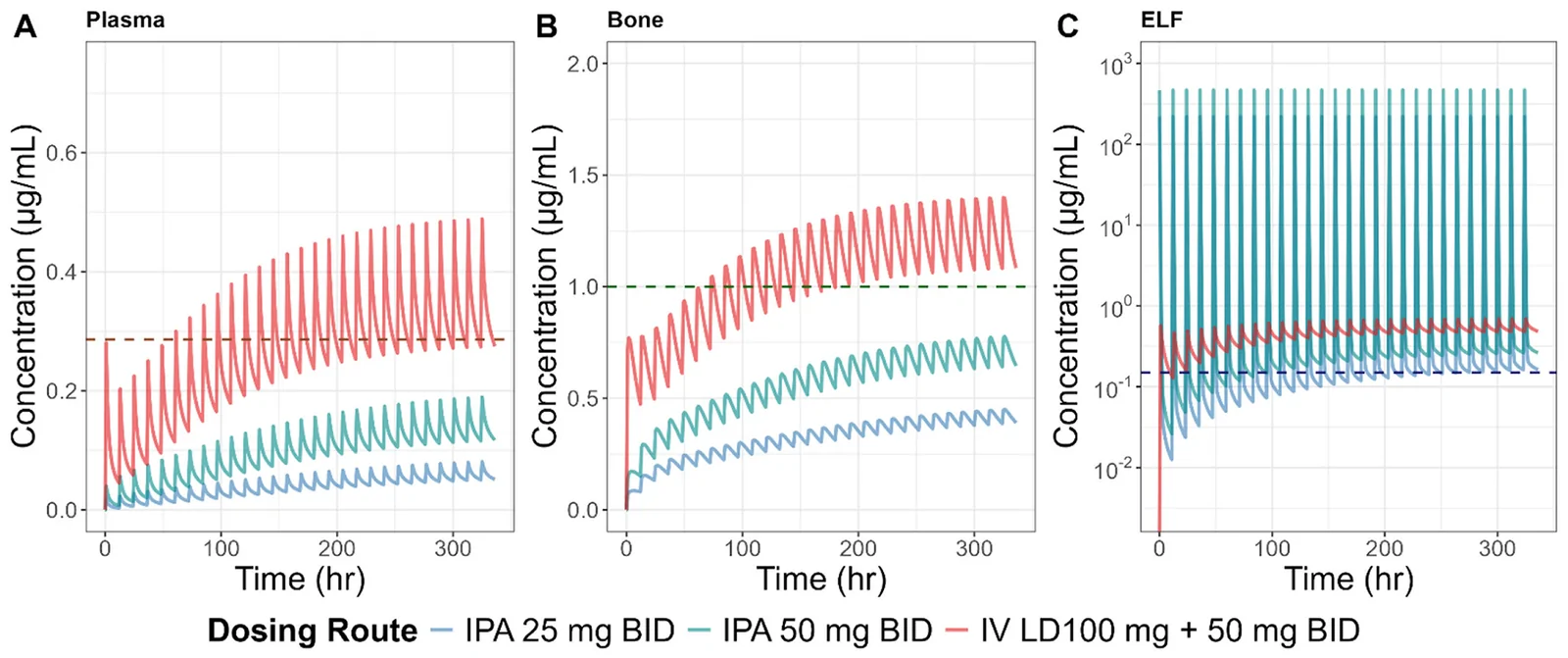
Comparison of longitudinal TGC concentration-time profiles in plasma **(A)**, bone **(B)**, and ELF **(C)** following IPA and IV administration. The horizontal dashed lines represent in **(A)** the average plasma concentration at steady state (C_avg.ss.pl_) derived from the gastrointestinal adverse event threshold (AUC_0 − 24.*ss*.*pl*_ = 6.87 mg·h/L), in **(B)** the bone safety threshold, defined as the steady-state bone trough concentration (C_trough.ss.bn_) simulated from the rat PBPK model, and in **(C)** the the steady-state trough ELF concentration (C_trough.ss.elf_) derived from the clinically efficacious dose (25 mg BID IPA), respectively.

**Figure 4:**
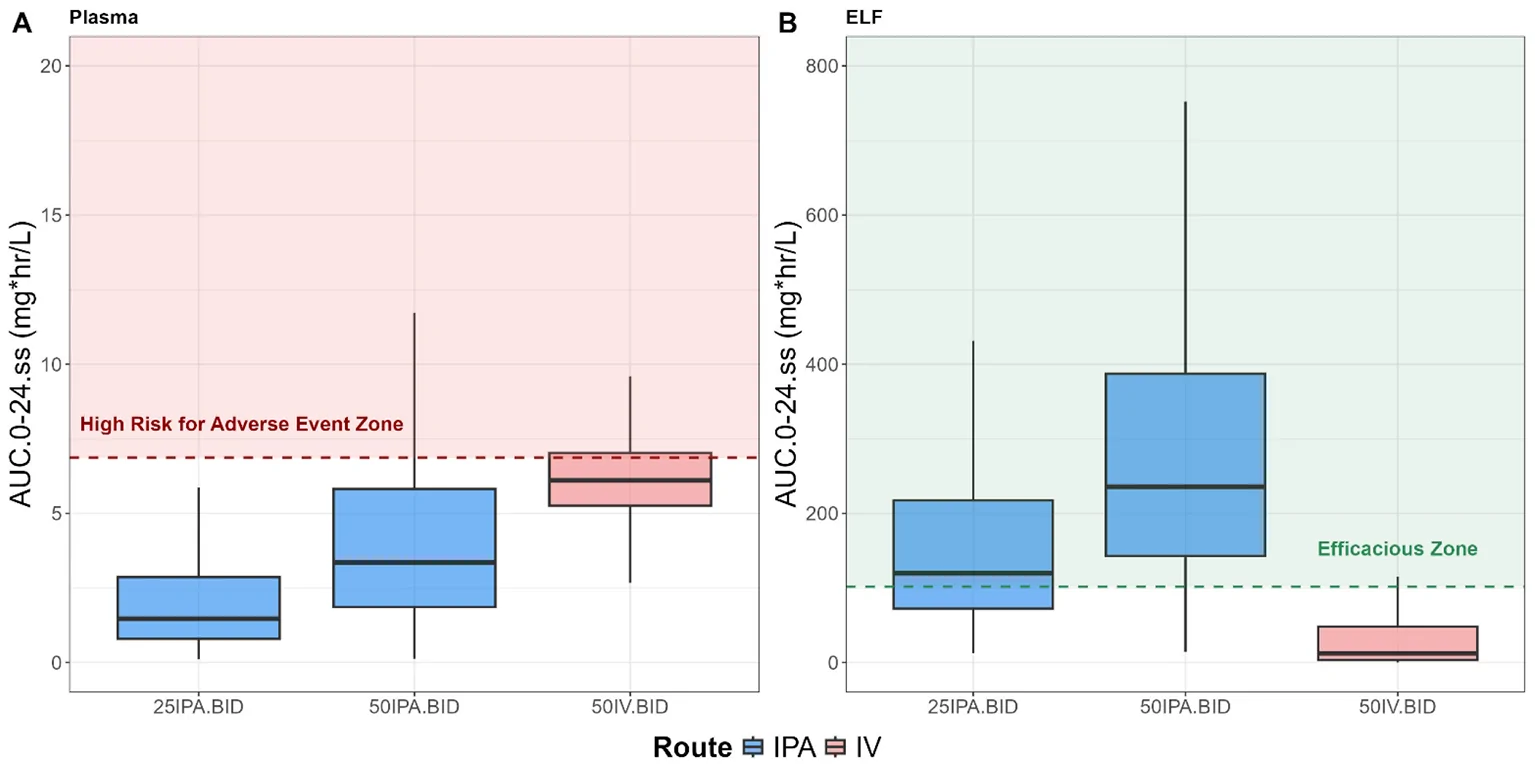
Comparison of relative exposures (AUC_0 − 24, *ss*_) in plasma **(A)** and ELF **(B)** following IV and IPA administration of TGC. The blue boxplots in panels **(A)** and **(B)** represent plasma (AUC_0 − 24.*ss*.*pl*_) and ELF (AUC_0 − 24.*ss*.*elf*_) exposures derived from 1,000 replicates of model-based simulations for IPA administration, respectively. The red boxplot in panels A and B represent AUC_0 − 24.*ss*.*pl*_ for the standard TGC IV dosing regimen and CV% reported from the literature (29), and AUC_0 − 24.*ss*.*elf*_ calculated from the model-based simulation profile. The horizontal dashed lines shown in panels **(A)** and **(B)** represent the safety threshold based on AUC_0 − 24.*ss*.*pl*_ reported in the literature for gastrointestinal toxicity (11) and the ELF exposure (AUC_0 − 24.*ss*.*elf*_) associated with bacterial clearance observed in *in vivo* animal studies of *M. abscessus* infection following IPA administration (12), respectively. BID twice daily.

As shown in Figure 3A, the steady-state systemic exposure in plasma following repeated IPA administration was lower than that observed after IV administration of the same dose level, and also fell below the average steady-state plasma concentration (C_avg.ss.pl_) derived from the previously reported systemic exposure-based gastrointestinal adverse event threshold (AUC_0 − 24.ss.pl_ = 6.87 mg h/L) (Figure 4) (11). In Figure 3B, the bone concentration profiles simulated for repeated IV administration at the standard clinical dose exceeded the bone safety threshold, which was defined as a simulated steady-state trough bone concentration (C_trough.ss.bn_) in rats administered with 20 mg/kg IV, based on the rat PBPK model (Figure S1 in the Supplementary material). In contrast, IPA administration with both regimens was predicted to result in lower steady-state bone concentrations, remaining below this putative adverse event threshold. Figure 3C demonstrates that ELF concentrations achieved after IPA administration result in dramatically higher peak levels compared to IV dosing due to the direct deposition of the drug into the pulmonary compartment. However, the ELF trough concentrations (C_trough.ss.elf_) were slightly higher under IV compared to IPA dosing. This was likely a consequence of the reduced bioavailability after IPA compared to IV administration. The resulting exposure levels (AUC_0 − 24.*ss*_) for the three dose levels in plasma and ELF and their predicted distribution range based on 1,000 simulation replicates accounting for inter-individual variability are summarized in Figure 4.

### 3.4 Exploration of maximum doses and dosing intervals

**Figure 5:**
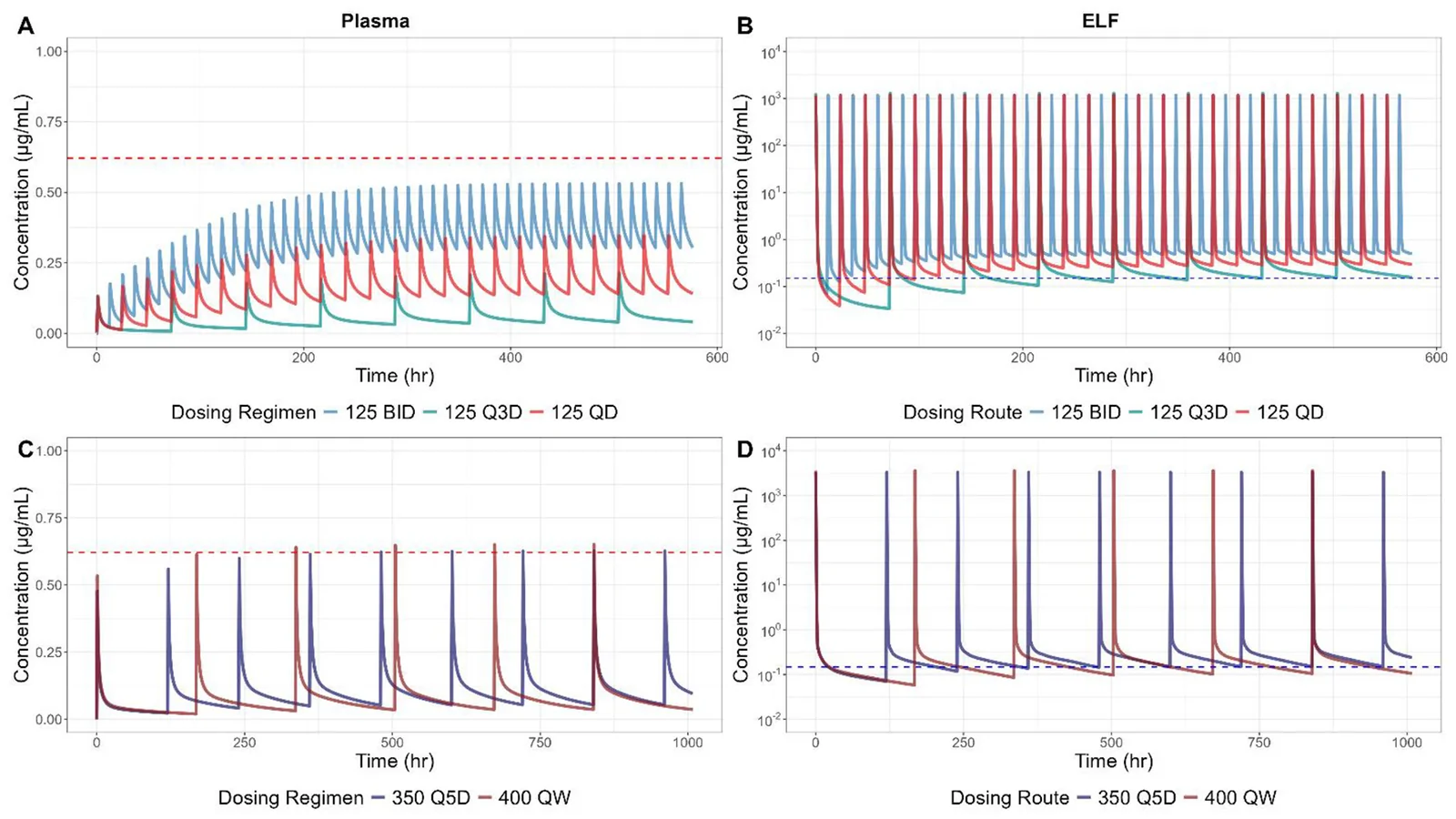
Comparison of simulated TGC concentration-time profiles in human plasma [panels **(A)** and **(C)]** and ELF [panels **(B)** and **(D)**] under various IPA dosing regimens, conducted using the developed PBPK model. The red dashed horizontal lines in panels **(A)** and **(C)** represent the steady-state maximum plasma concentration (C_max.ss.pl_), derived from systemic exposure-based safety threshold (AUC_0 − 24.*ss*.*pl*_ = 6.87 mg·h/L). The blue dashed horizontal lines in **(B)** and **(D)** indicate the steady-state trough ELF concentration (C_trough.ss.elf_) corresponding to the clinically efficacious dose of 25 mg twice daily via intrapulmonary aerosol delivery. Dose levels are provided in mg. BID trice daily, QD daily, Q3D every third day, Q5D every fifth day, QW once per week.

**Figure 6:**
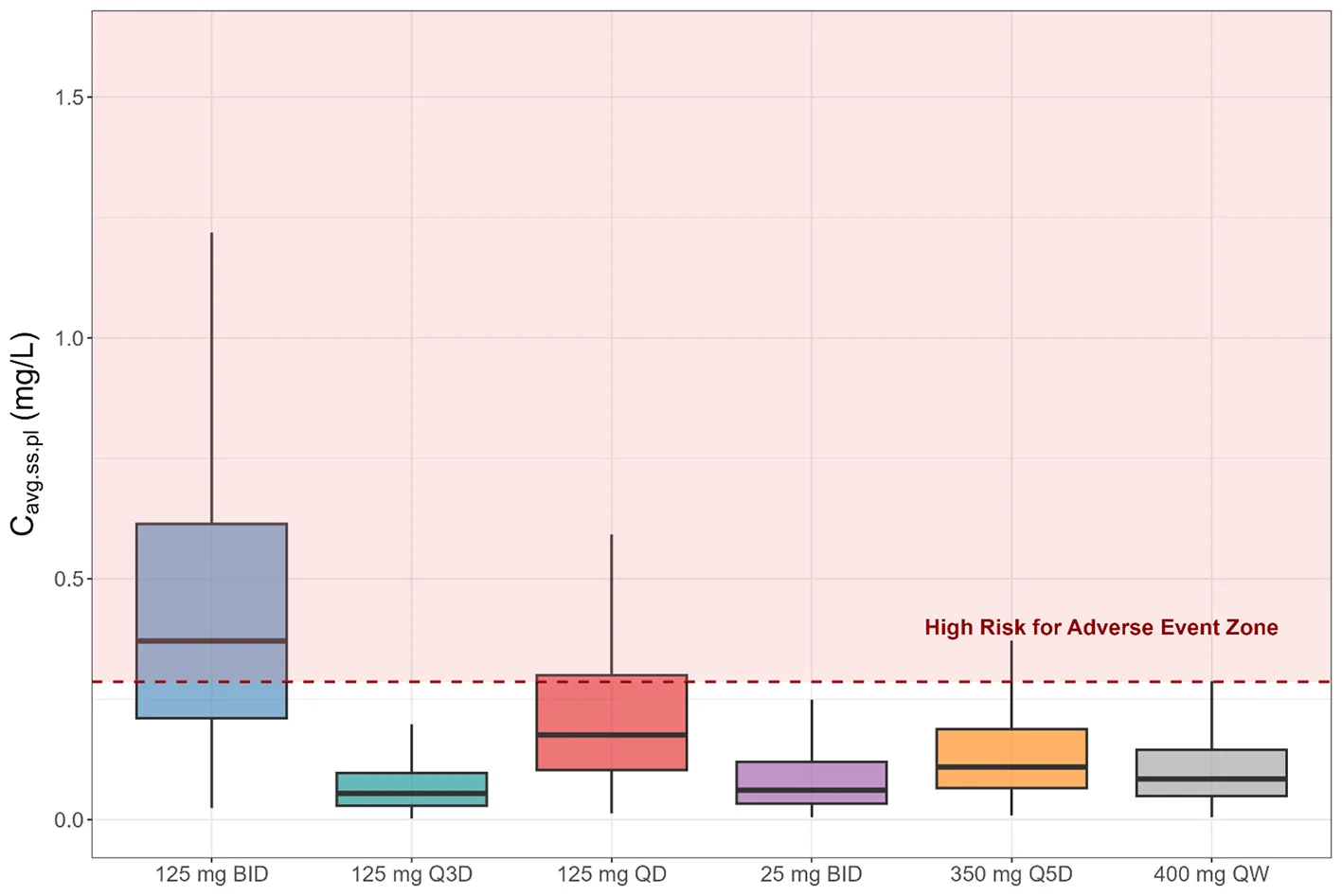
Comparison of simulated tigecycline exposures as average steady-state plasma concentrations (C_avg.ss.pl_) for various IPA dosing regimens. The boxplots represent the respective parameters and their interindividual variability derived from 1,000 replicates of model-based simulations for IPA administration. The dark red dotted horizontal line represents the literature reported systemic adverse event threshold derived from AUC_0 − 24.*ss*_ (11).

PBPK model-based simulations were performed to explore stepwise increases in exposure from the clinically efficacious dose (25 mg BID IPA) and to determine the maximum dose that could theoretically be administered safely by IPA based on two safety thresholds, the steady-state maximum plasma concentration C_max.ss.pl_ and the average steady-state plasma concentration C_avg.ss.pl_. When the safety criterion was defined as the C_max.ss.pl_ corresponding to the maximum systemic exposure-based adverse event threshold, it was predicted that the dose could be increased up to 125 mg with the same dosing interval (BID, Figure 5A). Similarly, when C_avg.ss.pl_ was used as the safety criterion, extending the dosing interval up to 3 days appeared to be a reasonable strategy from a safety perspective (Figure 6). Under the same conditions, the ELF concentrations achieved with each dosing regimen were predicted to remain above the C_trough.ss.elf_ of the clinically efficacious dose (25 mg BID) (Figure 5B). Assuming the maximum dosing interval that does not compromise patient adherence is one week (QW), the maximum IPA dose was estimated as approximately 400 mg when using C_max.ss.pl_ as the safety criteria. When applying a 5-day dosing interval, the maximum dose was predicted to be approximately 350 mg (Figures 5C, D). Although both dosing regimens maintained TGC exposure in the ELF above or close to the efficacious level, they failed to meet the safety threshold C_avg.ss.pl_ for gastrointestinal toxicity (Figure 6) when considering inter-individual variability. Thus, based on these considerations, 125 mg Q3D administered by the IPA route was identified as the most suitable dosing regimen for minimizing dosing frequency without compromising the established efficacy and safety exposure thresholds.

## 4 Discussion

Emergent drug resistance remains one of the main challenges in antibiotic therapy (40). Bacteria, including *M. abscessus*, frequently have or develop resistance to antibacterial agents that allow a subset of the population to evade antibiotic effects (41), and therapeutically inadequate, low doses of antibiotics have an increased probability of selecting for less susceptible bacterial populations, thereby conferring a high level of resistance in the overall bacterial population (42). To minimize the selective survival of resistant populations, it is important to induce extensive bacterial killing at the early stage of treatment and subsequently eliminate the less susceptible subpopulations that survive the initial exposure (43, 44). A common prerequisite for both, maximizing the proportion of bacteria that can be eradicated and for eliminating potentially resistant fractions, is to maintain optimally high local antibiotic drug concentrations at the site of infection. Therefore, localized drug delivery through IPA administration maximizing TGC concentrations at the site of action in pulmonary *Mab* infections represents a theoretically promising therapeutic strategy for suppressing the emergence of bacterial resistance.

In practice, several studies have demonstrated that exposure to subinhibitory concentrations of antibiotics can promote stepwise resistance evolution (45), whereas short-term treatment with high antibiotic concentrations is usually more effective at minimizing emergent resistance than prolonged exposure to low concentrations (46). These findings underscore the critical role of peak drug concentrations in suppressing the probability of resistance development, beyond the overall drug exposure. In addition, considering that biofilm formation by many bacteria, including *M. abscessus*, may impede the delivery of antibiotics at bactericidal concentrations (47, 48), treatment strategies that secure high drug concentrations at the site of infection are a promising strategy to overcome this barrier and achieve complete bacterial eradication.

The therapeutic viability of the otherwise conceptual application of TGC by IPA administration in pulmonary *M. abscessus* infection has recently been confirmed through a clinical case report (13). Local drug delivery through IPA leads to significantly higher drug exposure at the site of pulmonary infection compared to IV administration. However, the remaining key question is “what is the optimal IPA dose of TGC in humans that ensures both safety and patient adherence while at least delivering the effective exposure suggested by the recent clinical case report?” To answer this question, it was necessary to establish the relationship between drug exposure and adverse events, define the minimum effective exposure in the ELF as primary site of drug action, and, most importantly, develop a reliable method to accurately predict TGC exposure following IPA administration in humans which has not been reported to date.

Although clinical trials offer the most direct and reliable method to quantify drug exposure, a model-based approach was adopted as an alternative and first step to predict human exposure following IPA dosing with TGC. It should be noted that adopting a model-based approach reduces the risk and expense of entering clinical trials with poor estimates of doses required for efficacy and safety. A PBPK modeling framework was developed for TGC based on experimental and literature derived plasma and tissue exposure data in rodents after administration of IV, SC and IPA dosing and subsequently scaled to humans. Given that TGC is an approved drug with established PK profiles and available organ-specific exposure data in humans after IV administration, the key model parameters could be calibrated using this information during this scaling process. This step facilitated the reduction of uncertainty in the exposure profiles predicted by the human PBPK model. Furthermore, exposure safety thresholds previously reported in human studies were incorporated as reference points, providing a rationale for dosing regimen selection. While the target site-specific exposure following IPA doses was simulated using the established PBPK model and utilized the predicted trough concentration at steady state as minimum effective concentration needed to be maintained, it should be acknowledged that the exposure metric associated with minimum antibacterial efficacy may require further evaluation when additional clinical and experimental data become available for inhaled TGC.

As shown in Figures 3A, 4A, IPA administration at 50 mg BID resulted in lower systemic exposure at steady state compared to standard, approved IV administration at the same dose. Notably, plasma exposure of the 25 mg BID IPA regimen, which demonstrated clinical efficacy in the case report, was approximately 6-fold lower than that obtained with the IV regimen, supporting the rationale that IPA may reduce the likelihood of adverse events while maintaining therapeutic efficacy. Simulation for IV administration of the standard clinical dose (50 mg BID) resulted in TGC concentrations in bone that exceeded the safety threshold, which was defined by the rat PBPK model-based prediction of C_trough.ss.bn_ following a 20 mg/kg dose associated with toxicity (Figure 4B). This observation aligned with bone-associated adverse events listed in the drug label for TGC, indicating that the qualified rat and human PBPK-based modeling approach may provide meaningful insights on drug exposures in organs and tissues usually not monitored for drug exposures. Furthermore, bone exposure at steady state following IPA administration remained below the bone-specific safety threshold discussed above, supporting the notion that IPA dosing may offer a safer alternative to IV administration. With respect to the infection site, the peak concentration in ELF (Figure 4B) was predicted to be higher with IPA compared to the standard IV regimen, suggesting a potentially superior efficacy profile favorable to overcoming acquired antibiotic resistance and barrier effects from biofilm formation. The need for higher exposure is further supported by recent studies suggesting that previous failures of IV-based TGC therapy in critically ill patients with severe *Mab* infections were likely attributable to inadequately low standard dosing without considering susceptibility of the pathogen (49–51).

Although the only study investigating PK/PD indices for TGC against *M. abscessus* has utilized a hollow-fiber infection model (HFIM) (52), the AUC/MIC ratio is generally recognized as a predictive marker of efficacy for TGC across various infectious diseases, including pneumonia, intra-abdominal infections, and skin and soft tissue infections (49, 53, 54). Therefore, AUC seems to be a meaningful metric to compare dosing regimens for their potential for therapeutic efficacy. The model-based AUC_0 − 24.*ss*.*pl*_ predictions for each dosing regimen in our study are depicted in Figure 4A and compared to the known gastrointestinal adverse event threshold (11). The model-predicted median AUC_0 − 24.*ss*.*pl*_ and its distribution for the standard IV dosing regimen of 50 mg BID (29) approached the adverse event threshold and slightly overlapped with it, which is in line with literature findings (11). In contrast, the median AUC_0 − 24.*ss*.*pl*_ predicted for the IPA regimens were remarkably lower than the adverse event threshold. Notably, even the upper bound of predicted AUC_0 − 24.*ss*.*pl*_ for the 25 mg IPA group fell below the adverse event threshold. Similarly, the exposures simulated for the effect site (AUC_0 − 24.*ss*.*elf*_), shown in Figure 4B, were substantially higher for IPA compared to IV. Moreover, these ELF exposures exceeded the average exposure levels at steady-state (AUC_0 − 24.*ss*.*elf*_) observed in *in vivo* animal models at doses that demonstrated dramatic bacterial killing efficacy against *M. abscessus* (12). Taken together, these findings imply a greater level of safety and efficacy of IPA compared to IV administration in humans at the corresponding dose levels.

The maximum possible dose based on the C_max.ss.pl_, derived from the AUC_0 − 24.*ss*.*pl*_-based gastrointestinal adverse event threshold was also explored. From the model-based simulation results shown in Figure 5A, a dose of 125 mg BID was estimated to be the maximum IPA dose that does not compromise drug safety. However, the C_avg.ss.pl_, corresponding to 125 mg BID exceeded the adverse event threshold (Figure 6), suggesting that reducing the overall drug exposure by extending the dosing interval may be necessary to maximize IPA dosing that can be safely administered. When the dosing interval was extended to every three days (Q3D), the model predicted that C_avg.ss.pl_, of the Q3D regimen remained below the conservative level derived from the AUC_0 − 24.*ss*.*pl*_-based adverse event threshold. The median simulated C_avg.ss.pl_ for the 125 mg Q3D was comparable to that of clinically efficacious IPA dose (i.e., 25 mg BID) as shown in Figure 6. Notably, C_trough.ss.elf_ still remained above the level achieved by the same clinically efficacious IPA dosing regimen (Figure 5B). Therefore, if maximizing the IPA dose is intended, the 125 mg Q3D IPA regimen, based on the simulation results, is expected to exhibit substantially higher concentrations at the site of infection and lower plasma exposures (C_max.ss.pl_ and C_avg.ss.pl_). This outcome may offer a favorable balance of efficacy and safety compared to both, the 25 mg IPA BID and the 50 mg IV BID regimens. Similarly, the 350 mg Q5D and 400 mg QW regimens may also meet some of these criteria. However, considering the maximum deliverable dose of dry powder inhalation in humans (55), these options are deemed practically unfeasible for IPA dosing.

Although IPA administration of TGC is expected to meaningfully enhance efficacy, the potential risks associated with extremely high drug concentrations in the lungs, particularly when exceeding the clinically tested dose of 25 mg BID, remain unpredictable due to the lack of relevant safety data. Therefore, additional preclinical toxicology studies are warranted to assess the potential risk at elevated exposure levels in pulmonary tissues. Moreover, both of the exposure-based safety thresholds which have been associated with an increased incidence of adverse events, as well as the efficacy of IPA administration of TGC are currently only supported by limited clinical reports (13), highlighting the need for further accumulation of real-world clinical evidence and/or for dedicated clinical studies to confirm the appropriateness of the dosing regimens suggested by the presented model-based approach with regard to efficacy and safety. As demonstrated by the available clinical case study for inhaled tigecycline (13), it would be expected that the clinical use against *M. abscessus* infection would be in combination with other established antibiotics used in this condition (56).

As with most inhalation products available on the market, dry-powder formulations are likely to be prioritized for the clinical development of inhaled TGC, given its chemical instability in aqueous solution. However, due to limitations in experimentally evaluating powder-based inhalation formulations *in vivo* in rodents, model scale-up in the present work was performed using preclinical data generated with saline-based dosing solutions administered through IPA, under conditions comparable to the clinical case study reported by Pedersen et al. (13). Therefore, unlike the aerosol-based administration of solutions discussed in this manuscript, further investigation into the impact of formulation characteristics on the pharmacokinetics of TGC will be essential during product development for dry-powder based formulations, as they can help sustain exposure at the site of action and further reduce unnecessarily high exposures in local tissues and the systemic circulation. We have previously reported on a spray-dried tigecycline formulation for potential human use with a mass median aerodynamic diameter of 2.57 to 2.62 μm and a large fraction of around 70% in the respirable particle size range of 1–5 μm (57). Nevertheless, the model-based dosing recommendations proposed in this study, guided by orthogonal exposure thresholds for safety and efficacy, provide a scientifically informed basis for the feasibility assessment of IPA dosing of TGC in humans and provide guidance for clinical dosing regimen selection for future clinical trials using aerosol-based pulmonary delivery.

The model-based approach presented in this study provides a solid framework for proposing new therapeutic strategies during drug repositioning, such as optimizing the route of administration to balance efficacy and safety, particularly for compounds with strong efficacy but a narrow therapeutic window. In terms of scalability, the proposed mathematical model structure is also expected to serve as a foundation for translating human target site exposure into actual clinical efficacy through structural integration with various model components that describe exposure-response relationships including clinical susceptibility and/or the quantitative effects of covariates on drug efficacy under pathological conditions.

## Data Availability

The raw data supporting the conclusions of this article will be made available by the authors, without undue reservation.
