# Supplementary material for: Dose optimization of inhaled tigecycline in humans to overcome inherent adverse events and maximize bacterial clearance using a physiologically-based pharmacokinetic modeling approach
Source: Front Tuberc. 2026 Mar 26;4:1762713. doi: 10.3389/ftubr.2026.1762713 (PMC13225866; doi:10.3389/ftubr.2026.1762713)
Supplement: Supplementary file 1 [file Data_Sheet_1.pdf]

Supplementary Material

To

Dose Optimization of Inhaled Tigecycline in Humans to Overcome Inherent Adverse Events and Maximize Bacterial Clearance Using a PBPK Modeling Approach

Hyunseo Park, Amarinder Singh, Ashish Srivastava, Bhargavi Thalluri, Christelle J. Mathieu, Paridhi Gupta, Camron M. Pearce, Malik Zohaib Ali, Ilham M. Alshiraihi, Sara E. Maloney Norcross, Anthony J. Hickey, Mercedes Gonzalez-Juarrero, Bernd Meibohm

Table S1. Physiological parameters of mouse, rat and human used for physiologically-based pharmacokinetic model development.

Types	Parameters	Values			Unit	Reference
		Mouse	Rat	Human		
**Body weight**		0.025	0.25	70	kg	-
**Hematocrit**		0.45	0.46	0.44		Davies B. et al.(1)
**Blood flow**	Cardiac Output*	0.570560419	2.691955516	218.4	L/hr	Brown R. et al.(2) Shah D. et al.(3) Davies B. et al.(1)
	Lung	0.570560419	2.691955516	218.4	L/hr	
	Kidney	0.051920998	0.244967952	38.22	L/hr	
	Hepatic artery	0.011411208	0.05383911	10.0464	L/hr	
	Spleen	0.006418805	0.0302845	3.003	L/hr	
	GI tract	0.064501855	0.304325571	32.85828	L/hr	
	Pancreas	0.009528359	0.044955657	3.66912	L/hr	
	Liver	0.091860227	0.433404838	49.5768	L/hr	
	Bone	0.069608371	0.328418573	9.1728	L/hr	
	Adipose	0.039939229	0.188436886	11.3568	L/hr	
	Muscle	0.090719107	0.428020927	41.7144	L/hr	
	Brain	0.018828494	0.088834532	24.8976	L/hr	
	Heart	0.037656988	0.177669064	8.736	L/hr	
	Others	0.170027005	0.802202744	34.7256	L/hr	
**Organ/Tissue Volume**	Venous Plasma†	0.000876563	0.0054675	2.1756	L	Brown R. et al.(2) Davies B. et al.(1) Shao J & Hochhaus et al.(4)
	Arterial Plasma†	0.000292188	0.0018225	0.7252	L	
	Lung	0.0001825	0.00125	0.532	L	
	ELF	0.0000164	0.000164	0.04592	L	
	Kidney	0.0004175	0.001825	0.308	L	
	Spleen	0.0000875	0.0005	0.182	L	
	GI tract	0.001055	0.00675	1.197	L	
	Pancreas	0.00015	0.0008	0.098	L	
	Liver	0.0013725	0.00915	1.799	L	
	Bone	0.00265	0.0265	7.852797518	L	
	Adipose	0.00175	0.0175	16.36899563	L	
	Muscle	0.0096	0.101075	28	L	
	Brain	0.0004125	0.001425	1.4	L	
	Heart	0.000125	0.000825	0.329	L	
	Others	0.00602875	0.07511	9.032406849	L	

*Cardiac output adjusted by hematocrit of each species.

†The volume of arterial and venous blood were defined as 25 and 75 % of the intravascular spaces occupied by whole blood vessels and converted to plasma volume of respective compartments(5).

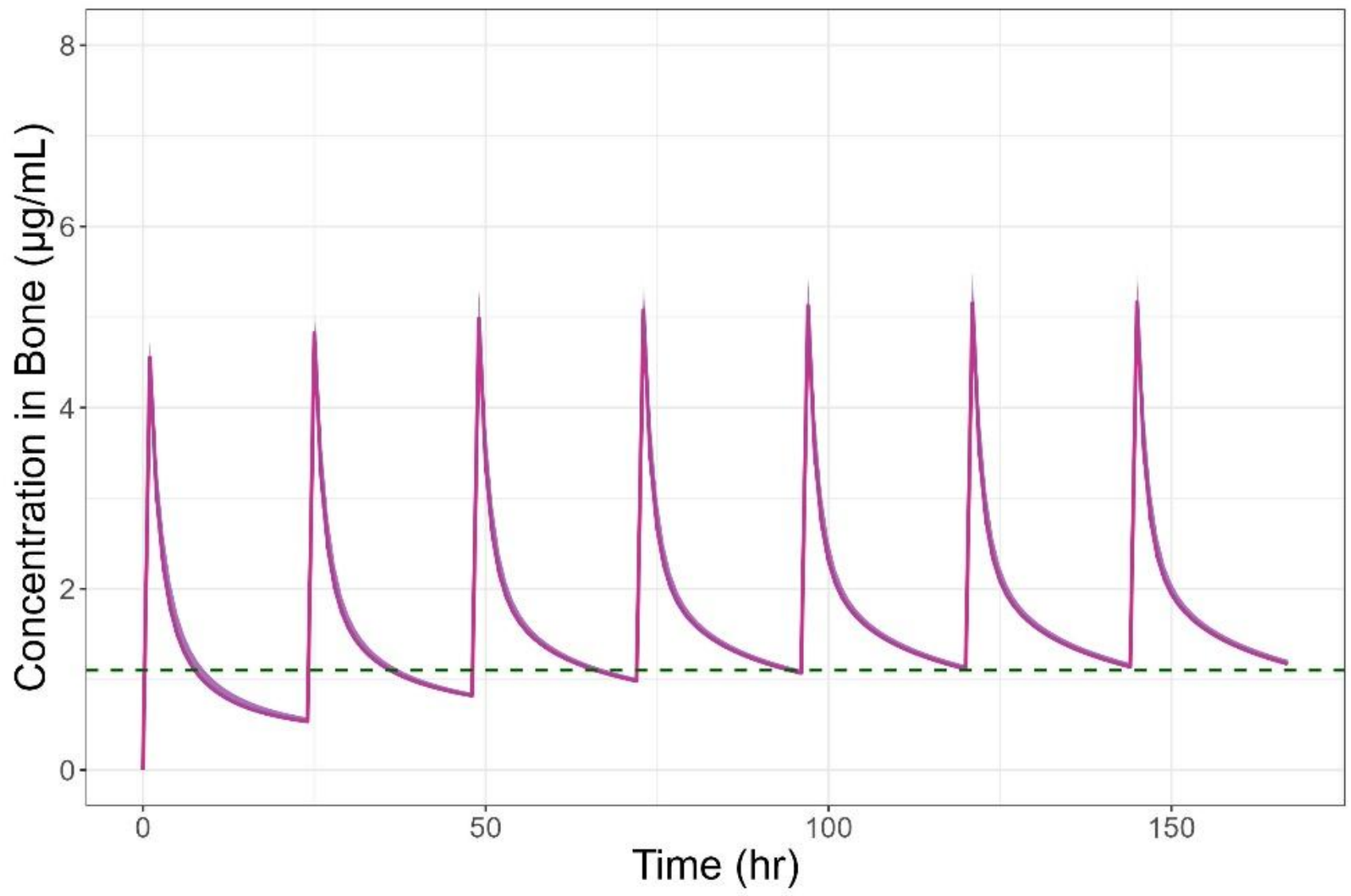

Figure S1. Rat PBPK model-based simulated concentration profiles of TGC in bone following 20 mg/kg IV administration, a dose reported to induce bone discoloration (6). The horizonal dotted line represents the steady-state trough concentration ($C_{trough.ss.bn}$) simulated from the rat PBPK model.

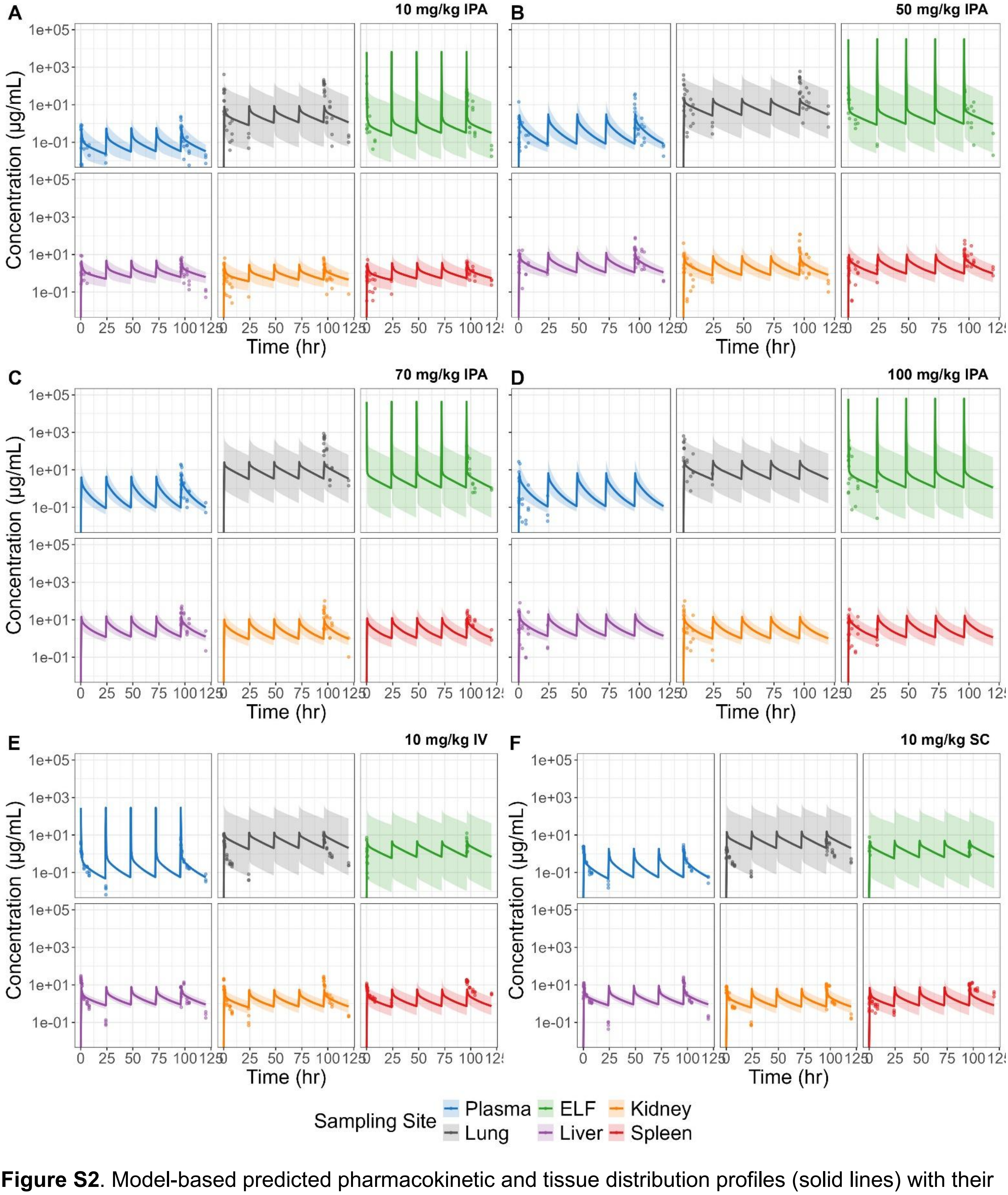

Figure S2. Model-based predicted pharmacokinetic and tissue distribution profiles (solid lines) with their 95% confidence intervals (corresponding color-shaded areas), compared with experimentally observed values (dots) from *in vivo* mouse studies.

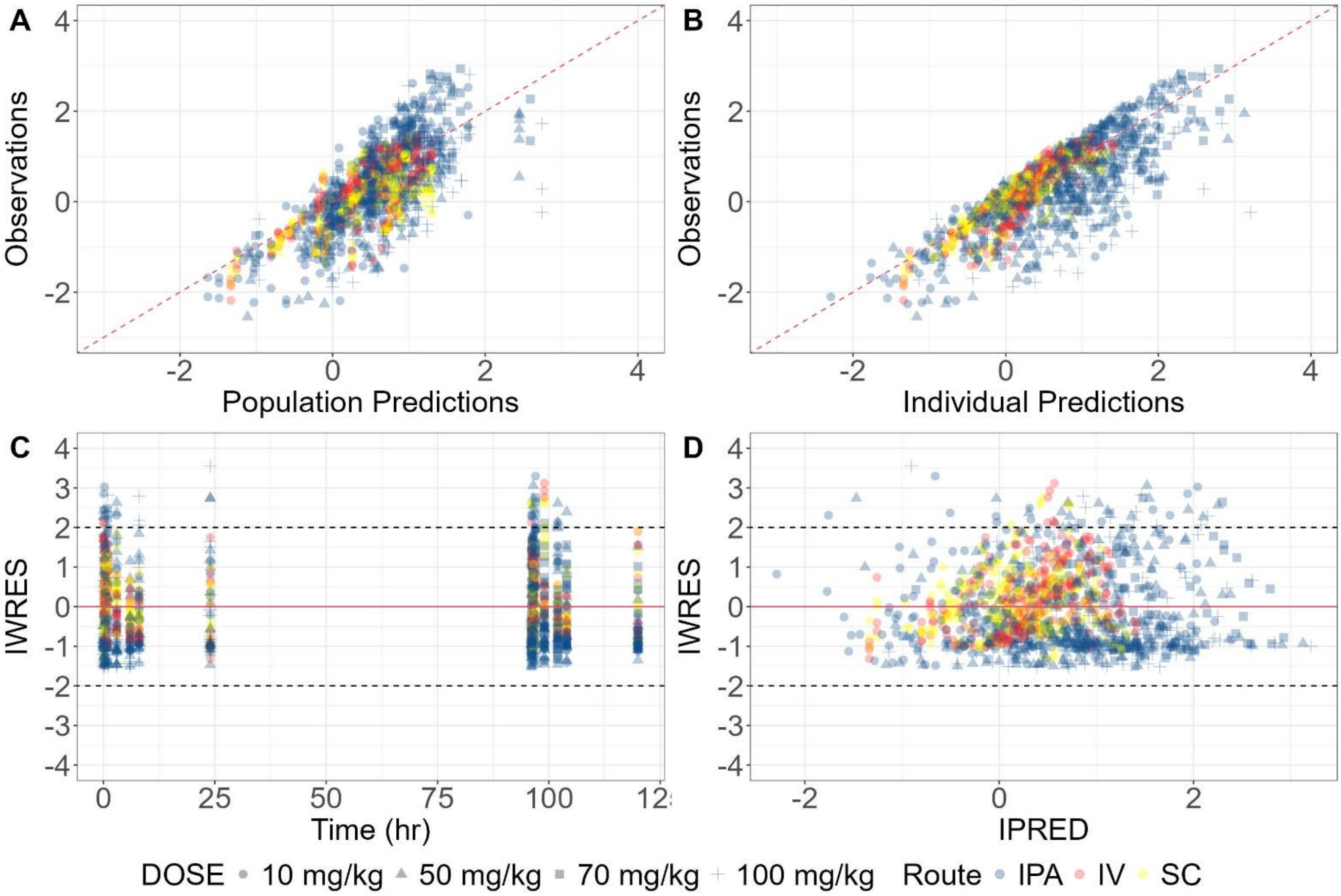

Figure S3. Model diagnostic plots for the mouse PBPK model: Observed versus population- (A) or individual- (B) predicted concentration; residual distributions versus time (C) and individually predicted concentration (D).

Model-Related Equations

Application of atypical protein binding of TGC

TGC exhibits concentration-dependent atypical plasma protein binding characteristics (7-9), which significantly influence *in vivo* distribution. Therefore, the effect of the concentration-dependent unbound fraction on the $K_{p.organ}$ was quantitatively incorporated into the model. Considering that TGC is a basic compound, the K_{pu} values for organs involved in PBPK model development were calculated using the following equation(10, 11).

$$K_{pu.organ} = f_{ew} + \frac{1+10^{pK_{a.b}-pH_{iw}}}{1+10^{pK_{a.b}-pH_p}} \cdot f_{iw} + \frac{K_{AP} \cdot [AP] \cdot 10^{pK_{a.b}-pH_{iw}}}{1+10^{pK_{a.b}-pH_{iw}}} + P \cdot f_{nl} + \frac{(0.3 \cdot P+0.7) \cdot f_{npl}}{1+10^{pK_{a.b}-pH_p}} \qquad \text{Eq.S1}$$

where f_{ew}, f_{iw}, f_{nl} and f_{npl} and are the fractional volume of extracellular water, intracellular water, neutral lipids and neutral phospholipids in the tissue, respectively; $[AP]$ is the concentration of acidic phospholipids in the tissue; K_{AP} is the association constant for acidic phospholipids in the tissue; $pK_{a.b}$ is the basic ionization constant.

Finally, the $K_{p.organ}$ for each organ were determined from the corresponding $K_{pu.organ}$ values and unbound fraction of TGC in plasma using Eq.S10 and Eq.S11 below.

$$K_{p.organ} = K_{pu.organ} \cdot f_{u.plasma} \qquad \text{Eq.S2}$$

$$f_{u.plasma} = (1 - \frac{I_{max} \cdot C_{plasma}}{IC_{50} + C_{plasma}}) \qquad \text{Eq. S3}$$

The atypical unbound fraction of TGC reported in the literature (9, 12) was described using an I_{max} model in Eq.11 and incorporated into the PBPK model to capture concentration-dependent tissue distribution through dynamic $K_{p.organ}$ values. I_{max} model was independently parameterized, with $I_{max}$and IC_{50}, values of 0.94 and 0.3 μg/mL, respectively (Data not shown). To ensure that the theoretically derived dynamic $K_{p.organ}$, which accounts for physicochemical properties, organ compositions, and atypical protein binding characteristics, adequately reflects the observed experimental values, an adjustment parameter $K_{p.organ}^{adj}$ was incorporated as shown in Eq.S12. The organ-specific values of this parameter were estimated through the model fitting process.

$$K_{p.organ}^{effective} = K_{pu.organ} \cdot f_{u.plasma} \cdot K_{p.organ}^{adj} \qquad \text{Eq.S4}$$

General Compartments

The concentration ratio between blood and plasma was assumed to follow a fixed blood-to-plasma partition ratio ($BP_{species}$) and to be independent of drug concentration(13). Accordingly, the model incorporated C_{plasma} (C_{blood}/BP_{mouse}) as an exposure metric, and organ blood flows (Q_{organ}) were adjusted using hematocrit of the mouse.

$$\frac{dC_{plasma}^{venous}}{dt} = (K_{a.sc} \cdot C_{depot}^{sc} \cdot V_{depot}^{sc} + \sum_{i=1}^{n-3}(Q_i \cdot \frac{C_i}{K_{p.i}^{effective}}) - Q_{lu} \cdot C_{plasma}^{venous})/V_{plasma}^{venous} \qquad \text{Eq.S5}$$

$$\frac{dC_{plasma}^{artery}}{dt} = (Q_{lung} \cdot \frac{C_{lung}}{K_{p.lung}^{effective}} - CO \cdot C_{plasma}^{artery})/V_{plasma}^{artery} \qquad \text{Eq.S6}$$

$$\frac{dC_{organ}}{dt} = (Q_{organ} \cdot (C_{plasma}^{artery} - \frac{C_{organ}}{K_{p.organ}^{effective}}))/V_{organ} \qquad \text{Eq.S7}$$

In the equations above, TGC concentration, volume, and blood flow of specific organs were represented as C_{site}, V_{site} and Q_{site}, respectively, with additional distinguishing details provided through superscripts. The subscript 'i' and superscript 'n' in Eq.S1 denote the index assigned to each organ and the total number of organs involved in the PBPK model, respectively. Since $K_{a.sc}$ represents the absorption rate constant from

the SC injection site, it was omitted for IV and IPA administration.

Kidney, Liver, Lung and ELF Compartment

For the liver, the input concentration corresponded to the mixed blood concentration entering from the hepatic portal vein and the venous outputs from the spleen and gastrointestinal (GI) tract. Hepatic ($CL_{hepatic}$) and renal clearance (CL_{renal}) of TGC was assumed to account for 65% and 35% of total clearance based on mass balance data reported in the literature(14). For the lungs, the input concentration was mixed venous return. The corresponding processes were mathematically described using the following equations.

$$\frac{dC_{kidney}}{dt} = (Q_{kidney} \cdot C_{plasma}^{artery} - (Q_{kidney} + CL_{renal}) \cdot \frac{C_{kidney}}{K_{p.kidney}^{effective}})/V_{kidney} \quad \text{Eq.S8}$$

$$\frac{dC_{liver}}{dt} = (Q_{portal} \cdot C_{plasma}^{artery} + Q_{spleen} \cdot \frac{C_{spleen}}{K_{p.spleen}^{effective}} + Q_{GI} \cdot \frac{C_{GI}}{K_{p.GI}^{effective}} + Q_{pancrease} \cdot \frac{C_{pancrease}}{K_{p.pancrease}^{effective}} - (Q_{liver} + CL_{hepatic}) \cdot \frac{C_{liver}}{K_{p.liver}})/V_{liver} \quad \text{Eq.S9}$$

$$\frac{dC_{lung}}{dt} = (Q_{lung} \cdot \left(C_{plasma}^{venous} - \frac{C_{lung}}{K_{p.lung}^{effective}}\right) - PS_{LE} \cdot (f_{u.lung} \cdot C_{lung} - f_{u.elf} \cdot C_{lung}^{elf}))/V_{lung} \quad \text{Eq.S10}$$

$$\frac{dC_{lung}^{elf}}{dt} = (PS_{LE} \cdot (f_{u.lung} \cdot C_{lung} - f_{u.elf} \cdot C_{lung}^{elf}))/V_{lung}^{elf} \quad \text{Eq.S11}$$

$$C_{lung}^{TOTAL} = (C_{lung} \cdot V_{lung} + C_{lung}^{elf} \cdot V_{lung}^{elf})/(V_{lung} + V_{lung}^{elf}) \quad \text{Eq.S12}$$

The transfer of TGC between the extravascular and the ELF region, as well as the reverse process, was described by the intercompartmental clearance term, PS_{LE}. Only the unbound fraction of TGC in lung parenchymal tissue ($f_{u.lung}$) was assumed to be available for transfer into the ELF region. Conversely, for the reverse transfer, the unbound fraction in ELF ($f_{u.elf}$) was assumed to be 1, considering negligible albumin content – the major binding protein of TGC – in ELF compared to plasma(15, 16).

MIxtran Model Code

```
DESCRIPTION:

[LONGITUDINAL]
input={ka, PS_le, Kp_li_adj, Kp_sp_adj, Kp_ki_adj, Kp_lu_adj, Kp_oth, fu_lu, F1}

PK:
BW = 0.025   ; kg
v_bl = BW*0.085*0.75    ; L, venous blood vol.
v_sc = 0.00005    ; L, administered vol.
v_lu_elf = BW/0.25*0.000164 ; converted from rat value 0.164 mL using AS (Surface area of central and pheriheral lung = 75 and 6357 cm2, and thickness of lining fluid layer = 5 and 0.2 um), EJPS 2021 Shao J & Hochhaus et al.

F=1 ; based on Bioavailability of compound after SC

mo_bp = 1     ; ***** mouse b/p ratio, changing from 1.1 to 0.6 from 0.1 ug/mL to 50 ug/mL *****
depot(adm = 1, target = c_pl, p=BW/v_bl/mo_bp)
depot(adm = 2, target = c_sc, p=F*BW/v_sc)
depot(adm = 3, target = c_lu_elf, p=F1*BW/v_lu_elf)

;;; mass          [=] mg
;;; volume        [=] L (or kg)
;;; concentration [=] ug/mL
;;; time          [=] hr

EQUATION:
odeType=stiff

;;;;;;;;;;;;;;;;;;;;;;;;;;;;;;;;;;;
;;;Physiological Parameters (P);;;
;;;;;;;;;;;;;;;;;;;;;;;;;;;;;;;;;;;

fu_pl = 0.0739*c_pl^(-0.373)   ; plasma unbound fraction from literature  ***** gradually decrease over the concentration range/Derendorf fup model *****
fu_pl_lu =(1-(0.94 * c_pl)/(0.3 + c_pl))
fu_elf = 1    ; ELF unbound fraction - assumed
mo_HCT = 0.45
```

;;; plasma flow : %CO

CO = 16.500*(1 - mo_HCT)*0.025^0.75 ; L/hr, mouse cardiac (plasma) output from literature (Brown etc.)

Q_bn = CO*0.122 ; assume that it is the same as rat's bone blood flow

Q_ad = CO*0.07

Q_ms = CO*0.159

Q_br = CO*0.033

Q_ht = CO*0.066

Q_lu = CO*1

Q_ki = CO*0.091

Q_hp = CO*0.02

Q_sp = CO*0.01125 ;Davies et al

Q_gi = CO*0.11305

Q_pc = CO*0.0167

Q_li = Q_hp + Q_sp + Q_gi + Q_pc ; sum is 0.1443, a bit different from literature value in Brown et al (Q_li = CO*0.161) maybe due to the Q of pancrease (0.017)

Q_oth = CO - (Q_ki + Q_li + Q_bn + Q_ad + Q_ms + Q_br + Q_ht)

;;

;;;Anatomical Characteristics (P) ;;;

;;

BW = 0.025 ; kg

;;; Volume (or weight) of organs (% of BW)

v_pl = BW*0.085*0.75*(1-0.45); HCT = 0.45

v_pl_a = BW*0.085*0.25*(1-0.45); Arterial Fraction of systemic circulation is about 25%

v_lu_elf = BW/0.25*0.000164 ; converted from rat value 0.164 mL using AS (Surface area of central and pheriheral lung = 75 and 6357 cm2, and thickness of lining fluid layer = 5 and 0.2 um), EJPS 2021 Shao J & Hochhaus et al.

v_lu = BW*0.0073

v_ki = BW*0.0167

v_li = BW*0.0549

v_sp = BW*0.0035

v_gi = BW*0.0422

v_pc = BW*0.006

v_bn = BW*0.106

v_ad = BW*0.07

```
v_ms = BW*0.384
v_br = BW*0.0165
v_ht = BW*0.005

v_oth = BW - v_pl - v_pl_a - v_lu - v_ki - v_li - v_sp - v_gi - v_bn - v_ad - v_ms - v_br - v_ht - v_pc


;;;;;;;;;;;;;;;;;;;;;;;;;;;;;;;;;;;;;;;;;;;
;;;Parameters empirically calculated (P);;;
;;;;;;;;;;;;;;;;;;;;;;;;;;;;;;;;;;;;;;;;;;;

;;;Kpu_tissue, calculated based on RR method
Kpu_ad = 18.40
Kpu_bn = 31.12
Kpu_br = 332.7
Kpu_gi = 111.4
Kpu_ht = 121.5
Kpu_ki = 169.4
Kpu_li = 150.8
Kpu_lu = 118.3
Kpu_ms = 283.1
Kpu_sp = 96.02
Kpu_pc = 96.02

Kp_ht =  fu_pl * Kpu_ht
Kp_br = fu_pl * Kpu_br
Kp_ms = fu_pl * Kpu_ms
Kp_gi = fu_pl * Kpu_gi
Kp_li = fu_pl * Kpu_li * Kp_li_adj
Kp_sp = fu_pl * Kpu_sp * Kp_sp_adj
Kp_ki = fu_pl * Kpu_ki * Kp_ki_adj
Kp_lu = fu_pl_lu * Kpu_lu * Kp_lu_adj
Kp_ad = fu_pl * Kpu_ad
Kp_bn = fu_pl * Kpu_bn
Kp_pc = fu_pl * Kpu_pc
;;;Kp_oth

;;;Metabolism
CL_T = 0.054     ; 0.054 L/hr, calculated from obs. data (iv plasma CLtotal = 2.16 L/hr/kg)
```

```
Fh = 0.65     ; from FDA Clin Pharm Rev & Mass balance study

Fr = 0.35     ; from FDA Clin Pharm Rev & Mass balance study

CL_H = CL_T*Fh      ;  have to assign CLt and Fh and Fr

CL_R = CL_T*Fr      ;  have to assign CLt and Fh and Fr


;;;;;;;;;;;;;;;;;;;;;;;;

;;;Initial Values (I);;;

;;;;;;;;;;;;;;;;;;;;;;;;

c_pl_0 = 0

c_pl_a_0 = 0

c_lu_0 = 0

;c_lu_vas_0 = 0

;c_lu_ti_0 = 0

c_lu_elf_0 = 0

c_ht_0 = 0

c_br_0 = 0

c_ms_0 = 0

c_ad_0 = 0

c_bn_0 = 0

c_gi_0 = 0

c_sp_0 = 0

c_pc_0 = 0

c_li_0 = 0

c_ki_0 = 0

c_oth_0 = 0

c_sc_0 = 0


;;;;;;;;;;;;;;;;;;;;;;;;;;;;;;;;;

;;;Differential Equation (ODE);;;

;;;;;;;;;;;;;;;;;;;;;;;;;;;;;;;;;

ddt_c_sc = -ka*c_sc


ddt_c_pl =  (ka*c_sc*v_sc + Q_ht*(c_ht/Kp_ht) + Q_br*(c_br/Kp_br) + Q_ms*(c_ms/Kp_ms) + Q_ad*(c_ad/Kp_ad) +
Q_bn*(c_bn/Kp_bn) + Q_li*(c_li/Kp_li) + Q_ki*(c_ki/Kp_ki) + Q_oth*(c_oth/Kp_oth) - Q_lu*(c_pl))/v_pl


ddt_c_pl_a = (Q_lu*c_lu/Kp_lu - CO*c_pl_a)/v_pl_a

ddt_c_lu = (Q_lu*(c_pl - c_lu/Kp_lu) - PS_le*(fu_lu*c_lu - fu_elf*c_lu_elf))/v_lu

ddt_c_lu_elf = (PS_le*(fu_lu*c_lu - fu_elf*c_lu_elf))/v_lu_elf ;added fu

c_lu_t = (c_lu*v_lu + c_lu_elf*v_lu_elf)/(v_lu + v_lu_elf)
```

```
ddt_c_ht = (Q_ht*(c_pl_a - c_ht/Kp_ht))/v_ht      ; Kp_ht calc, c_ht?
ddt_c_br = (Q_br*(c_pl_a - c_br/Kp_br))/v_br      ; Kp_br calc, c_br?
ddt_c_ms = (Q_ms*(c_pl_a - c_ms/Kp_ms))/v_ms      ; Kp_ms calc, c_ms?
ddt_c_ad = (Q_ad*(c_pl_a - c_ad/Kp_ad))/v_ad      ; Kp_ad calc, c_ad?
ddt_c_bn = (Q_bn*(c_pl_a - c_bn/Kp_bn))/v_bn      ; Kp_bn calc, c_bn?
ddt_c_gi = (Q_gi*(c_pl_a - c_gi/Kp_gi))/v_gi      ; Kp_gi calc, c_gi?
ddt_c_sp = (Q_sp*(c_pl_a - c_sp/Kp_sp))/v_sp      ; Kp_sp = estimate, c_sp = obs.value
ddt_c_pc = (Q_pc*(c_pl_a - c_pc/Kp_pc))/v_pc      ; Kp_pc calc, c_gi?
ddt_c_li = (Q_hp*c_pl_a + Q_sp*c_sp/Kp_sp + Q_gi*c_gi/Kp_gi + Q_pc*c_pc/Kp_pc - Q_li*c_li/Kp_li - CL_H*c_li/Kp_li)/v_li
ddt_c_ki = (Q_ki*(c_pl_a - c_ki/Kp_ki) - CL_R*c_ki/Kp_ki)/v_ki

ddt_c_oth = (Q_oth*(c_pl_a - c_oth/Kp_oth))/v_oth

; DVID / 1, 2, 3, 4, 5 = Plasma, Kidney, Liver, Lung, Spleen

OUTPUT:
output = {c_pl, c_ki, c_li, c_lu, c_sp, c_lu_elf, fu_pl}
```

References

1. Davies B, Morris T. Physiological parameters in laboratory animals and humans. Pharm Res. 1993;10(7):1093-5.

2. Brown RP, Delp MD, Lindstedt SL, Rhomberg LR, Beliles RP. Physiological parameter values for physiologically based pharmacokinetic models. Toxicol Ind Health. 1997;13(4):407-84.

3. Shah DK, Betts AM. Towards a platform PBPK model to characterize the plasma and tissue disposition of monoclonal antibodies in preclinical species and human. J Pharmacokinet Pharmacodyn. 2012;39(1):67-86.

4. Shao J, Wang Y, Hochhaus G. Semi-mechanistic PK/PD model to assess pulmonary targeting of beclomethasone dipropionate and its active metabolite. Eur J Pharm Sci. 2021;159:105699.

5. Hua J, Liu P, Kim T, Donahue M, Rane S, Chen JJ, et al. MRI techniques to measure arterial and venous cerebral blood volume. Neuroimage. 2019;187:17-31.

6. U.S. Food and Drug Administration. Pharmacology/Toxicology Review and Evaluation for Tygacil (tigecycline). FDA; 2005.

7. Deitchman AN, Singh RSP, Derendorf H. Nonlinear Protein Binding: Not What You Think. J Pharm Sci. 2018;107(7):1754-60.

8. Mukker JK, Singh RP, Derendorf H. Determination of atypical nonlinear plasma-protein-binding behavior of tigecycline using an in vitro microdialysis technique. J Pharm Sci. 2014;103(3):1013-9.

9. Singh RSP, Mukker JK, Drescher SK, Deitchman AN, Derendorf H. A need to revisit clinical breakpoints of tigecycline: effect of atypical non-linear plasma protein binding. Int J Antimicrob Agents. 2017;49(4):449-55.

10. Rodgers T, Leahy D, Rowland M. Physiologically based pharmacokinetic modeling 1: predicting the tissue distribution of moderate-to-strong bases. J Pharm Sci. 2005;94(6):1259-76.

11. Rodgers T, Rowland M. Physiologically based pharmacokinetic modelling 2: predicting the tissue distribution of acids, very weak bases, neutrals and zwitterions. J Pharm Sci. 2006;95(6):1238-57.

12. Bulik CC, Wiskirchen DE, Shepard A, Sutherland CA, Kuti JL, Nicolau DP. Tissue penetration and pharmacokinetics of tigecycline in diabetic patients with chronic wound infections described by using in vivo microdialysis. Antimicrob Agents Chemother. 2010;54(12):5209-13.

13. Chen Q, Tung EC, Ciccotto SL, Strauss JR, Ortiga R, Ramsay KA, et al. Effect of the anticoagulant ethylenediamine tetra-acetic acid (EDTA) on the estimation of pharmacokinetic parameters: A case study with tigecycline and ciprofloxacin. Xenobiotica. 2008;38(1):76-86.

14. Hoffmann M, DeMaio W, Jordan RA, Talaat R, Harper D, Speth J, et al. Metabolism, excretion, and pharmacokinetics of [14C]tigecycline, a first-in-class glycylcycline antibiotic, after intravenous infusion to healthy male subjects. Drug Metab Dispos. 2007;35(9):1543-53.

15. Rennard SI, Basset G, Lecossier D, O'Donnell KM, Pinkston P, Martin PG, et al. Estimation of volume of epithelial lining fluid recovered by lavage using urea as marker of dilution. J Appl Physiol (1985). 1986;60(2):532-8.

16. Stojanović SD, Janković SM, Matović ZD, Jakovljević IŽ, Jelić RM. Interaction between tigecycline and human serum albumin in aqueous solution. Monatshefte für Chemie - Chemical Monthly. 2015;146(2):399-409.
